# Connecting Older Adults With Mental Health Apps: A Survey of Provider App and Education Material Use

**DOI:** 10.1093/geroni/igab046.3135

**Published:** 2021-12-17

**Authors:** Priyanka Mehta, Chalise Carlson, Jason Anderson, Ana Alfaro, Erin Sakai, Christine Gould

**Affiliations:** 1 VA Palo Alto Health Care System, Palo Alo, California, United States; 2 VA Palo Alto Health Care System, Palo Alto, California, United States

## Abstract

Many older veterans have access to mobile devices and are interested in using apps for mental health self-management, but few have ever downloaded health apps. To address the need for awareness of and access to VA mental health apps, we developed patient educational materials aimed towards older (or novice) users of mobile devices. The present study explored health care providers’ and staff’s perceptions about use of mental health mobile applications (apps) with older veterans and examined potential utility of these patient educational materials. Requestors of mobile device education materials (N = 90) were surveyed when ordering materials and again 4 months later. Baseline and follow-up surveys assessed frequency of app recommendation, and comfort recommending apps. Baseline surveys examined perceived advantages of apps; follow-up surveys examined perceived utility of the educational materials. Descriptive statistics and qualitative analysis were conducted. Most requesters (68.5%) initially were not comfortable using apps, yet perceived many advantages to using apps and hoped materials could facilitate app use. At follow-up, requestors felt more comfortable recommending apps alongside our materials. Qualitative analysis revealed perceived advantages to using the education materials. The benefits of developing and disseminating educational materials for providers to share with older veterans helped support older veterans’ app use, and potentially increased providers’ comfort with and frequency of recommending apps to their older patients. Access to educational materials can mitigate discomfort among providers in recommending apps to older users and may bring about valuable discussions about apps which support mental health.

